# Restoration treatments enhance tree growth and alter climatic constraints during extreme drought

**DOI:** 10.1002/eap.3072

**Published:** 2024-12-03

**Authors:** Kyle C. Rodman, John B. Bradford, Alicia M. Formanack, Peter Z. Fulé, David W. Huffman, Thomas E. Kolb, Ana T. Miller‐ter Kuile, Donald P. Normandin, Kiona Ogle, Rory J. Pedersen, Daniel R. Schlaepfer, Michael T. Stoddard, Amy E. M. Waltz

**Affiliations:** ^1^ Ecological Restoration Institute Northern Arizona University Flagstaff Arizona USA; ^2^ US Geological Survey, Northwest Climate Adaptation Science Center Seattle Washington USA; ^3^ US Geological Survey, Southwest Biological Science Center Flagstaff Arizona USA; ^4^ School of Informatics, Computing, and Cyber Systems Northern Arizona University Flagstaff Arizona USA; ^5^ School of Forestry Northern Arizona University Flagstaff Arizona USA; ^6^ USDA Forest Service, Rocky Mountain Research Station Flagstaff Arizona USA; ^7^ Center for Adaptable Western Landscapes Northern Arizona University Flagstaff Arizona USA

**Keywords:** basal area increment, climate memory, dry forests, forest thinning, moisture stress, *Pinus ponderosa* var. *scopulorum*, prescribed fire, southwestern United States

## Abstract

The frequency and severity of drought events are predicted to increase due to anthropogenic climate change, with cascading effects across forested ecosystems. Management activities such as forest thinning and prescribed burning, which are often intended to mitigate fire hazard and restore ecosystem processes, may also help promote tree resistance to drought. However, it is unclear whether these treatments remain effective during the most severe drought conditions or whether their impacts differ across environmental gradients. We used tree‐ring data from a system of replicated, long‐term (>20 years) experiments in the southwestern United States to evaluate the effects of forest restoration treatments (i.e., evidence‐based thinning and burning) on annual growth rates (i.e., basal area increment; BAI) of ponderosa pine (*Pinus ponderosa*), a broadly distributed and heavily managed species in western North America. The study sites were established at the onset of the most extreme drought event in at least 1200 years and span much of the climatic niche of Rocky Mountain ponderosa pine. Across sites, tree‐level BAI increased due to treatment, where trees in treated units grew 133.1% faster than trees in paired, untreated units. Likewise, trees in treated units grew an average of 85.6% faster than their pre‐treatment baseline levels (1985 to ca. 2000), despite warm, dry conditions in the post‐treatment period (ca. 2000–2018). Variation in the local competitive environment promoted variation in BAI, and larger trees were the fastest‐growing individuals, irrespective of treatment. Tree thinning and prescribed fire altered the climatic constraints on growth, decreasing the effects of belowground moisture availability and increasing the effects of atmospheric evaporative demand over multi‐year timescales. Our results illustrate that restoration treatments can enhance tree‐level growth across sites spanning ponderosa pine's climatic niche, even during recent, extreme drought events. However, shifting climatic constraints, combined with predicted increases in evaporative demand in the southwestern United States, suggest that the beneficial effects of such treatments on tree growth may wane over the upcoming decades.

## INTRODUCTION

Recent increases in extreme drought pose a major threat to Earth's plant communities (Hartmann et al., 2022; Moss et al., 2024). In forested ecosystems, droughts caused by increasing temperatures and altered precipitation regimes have triggered widespread tree mortality (Allen et al., 2010; Sommerfeld et al., 2018), reduced primary productivity (Potter et al., 2024), and altered community structure and composition (Batllori et al., 2020). Such events may directly harm trees through xylem cavitation and embolism (i.e., disruption of hydraulic pathways within the tree), carbon starvation (i.e., a decline in available carbon reserves due to reduced photosynthesis during stress), and an increased vulnerability to biotic mortality agents (e.g., bark beetles; Coleoptera: Curculionidae: Scolytinae) (Adams et al., 2017; Kolb et al., 2016; Trugman et al., 2021). Droughts also indirectly influence forest dynamics by modifying inter‐ and intraspecific interactions among trees, and interactions between individuals and their environment (Clark et al., 2016). Together, these direct and indirect effects can have lasting impacts on processes such as tree growth, which, in turn, have major implications for global carbon dynamics (Anderegg et al., 2015; McDowell et al., 2020). However, many of Earth's forests are actively managed by humans (Grantham et al., 2020; Lesiv et al., 2022), and effective management may help mitigate the impacts of drought on forested ecosystems (Griscom et al., 2017). Thus, understanding the effects of management activities on drought resistance is critical to ensure that such actions promote forest ecosystem integrity in a warming world.

Forest management activities such as mechanical thinning (i.e., partial removal of trees within a stand) and prescribed burning (i.e., use of intentionally ignited fires) are being widely applied to restore ecological function in seasonally dry forest systems. In the United States, nearly 10,000 km^2^ year^−1^ of USDA (US Department of Agriculture) Forest Service lands are treated with mechanical thinning or prescribed fire, with expected increases in rates of treatment over upcoming years (Ager, 2022; Vaillant & Reinhardt, 2017). Many such treatments are intended to reduce fire hazard and restore patterns and processes in forest ecosystems where relatively frequent surface fire regimes have been interrupted (i.e., “forest restoration”; Hessburg et al., 2021; Stephens et al., 2021). Restoration treatments may also have associated co‐benefits such as increased forest resistance to drought, which may be considered in cost–benefit analyses and broader climate adaptation frameworks (e.g., Noel et al., 2023; Schuurman et al., 2022). Overall, thinning can ameliorate the effects of drought and enhance the growth of the remaining trees, although effects vary by taxa (Sohn et al., 2016) and differ across biophysical gradients (Bradford et al., 2021). Prescribed burning can improve drought resistance by reducing competition (van Mantgem et al., 2021) and promote resistance to biotic agents such as bark beetles by enhancing allocation to defensive compounds (Hood et al., 2015). On the other hand, low‐severity burning can cause nonlethal injuries that may reduce short‐term growth (Willson et al., 2024), disrupt physiological processes (Bär et al., 2019), and sometimes increase tree susceptibility to subsequent drought or bark beetle colonization (McHugh et al., 2003; Steel et al., 2021). Variation in the effects of thinning and burning treatments on drought resistance may be due to differences in local treatment implementation or inter‐ or intraspecific differences in tree response. Therefore, controlled experiments conducted across biophysical gradients (e.g., Schwilk et al., 2009; Stoddard et al., 2021) are needed to disentangle such effects.

**FIGURE 1:**
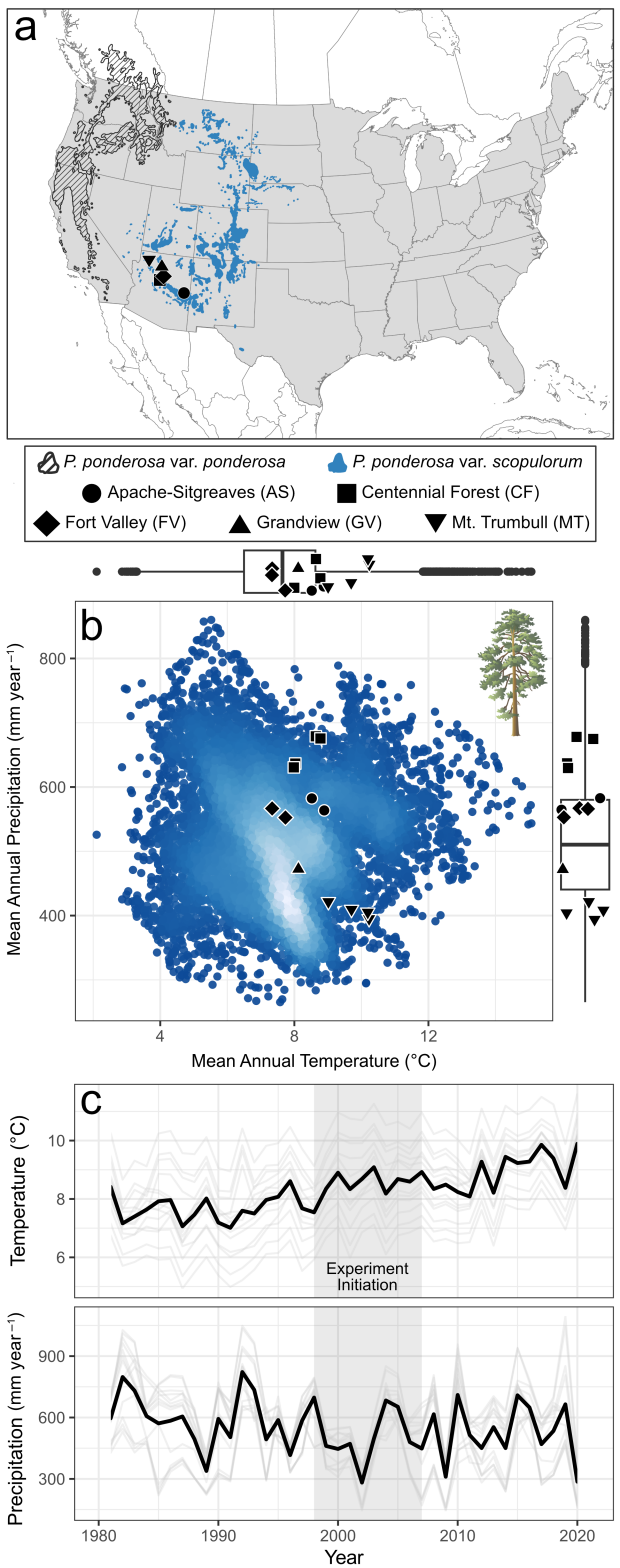
Locations of experimental sites in Arizona, USA, within (a) the broader range of ponderosa pine (*Pinus ponderosa*) in western North America (Little, 1971). Scatterplot and boxplots (b) show the average climate (from 1991 to 2020) (Thornton et al., 2021) in replicated experimental blocks within five sites (black shapes), relative to the climate range of the Rocky Mountain variety (*P. ponderosa* var. *scopulorum*) (Shinneman et al., 2016). In (c), trends in mean annual temperature and total annual precipitation are given for individual experimental blocks (gray lines) and the broader study area (black line). Ponderosa pine graphic in panel (b) was created by Kim Kraeer and Lucy Van Essen‐Fishman and obtained via the Integration and Application Network Media Library (ian.umces.edu/media‐library).

Ponderosa pine (*Pinus ponderosa* Douglas ex C.Lawson) is one of the most abundant and widely distributed tree species in North America (Little, 1971; Oswalt et al., 2019; Figure 1), and forests dominated by this species (i.e., “dry forests”) are more commonly managed using thinning and burning than many other forest types (Schoennagel & Nelson, 2011). Across the species' range, individual ponderosa pine trees are likely to respond differently to treatment due to ontogeny, inter‐ and intraspecific interactions, and broad‐scale environmental gradients. For example, small‐ to intermediate‐sized trees (i.e., <40 cm dbh) often show the greatest enhancements in growth following thinning (Fulé et al., 2022; Skov et al., 2005). In dense forests where fire has been excluded for several decades, higher levels of tree removal can provide a greater treatment effect (Andrews et al., 2020). Effects may also vary with pre‐treatment stand density and the location of a population within a species range. For example, ponderosa pine growth responses to climate differ across environmental gradients (Peltier & Ogle, 2019; Yocom et al., 2022). Theory (e.g., the stress gradient hypothesis; Bertness & Callaway, 1994) also suggests that facilitative interactions are more important on arid sites, whereas competition is more important in mesic sites. Thus, thinning and burning, which often promote drought resistance by reducing competition for resources (Kerhoulas et al., 2023; Tepley et al., 2020), might be expected to have the greatest effects in dense forest stands found in wetter areas (Gleason et al., 2017; Young et al., 2023). Understanding variation in ponderosa pine response to treatment will help prioritize management in locations with comparatively greater benefits and provide insight into underlying mechanisms.

We leveraged a long‐term (>20 years) network of replicated experiments in the southwestern United States, with sites spanning a broad climatic range (Figure 1), to evaluate the impacts of forest restoration treatments on ponderosa pine radial growth (i.e., annual basal area increment [BAI]) during the most extreme drought event in at least 1200 years (Williams et al., 2022). We focused specifically on radial growth because it can be accurately measured at annual to seasonal timescales, provides key insights into aboveground carbon storage (Babst et al., 2014), and is related to defensive allocation in ponderosa pine (Gonzalez et al., 2023; Kane & Kolb, 2010). We assessed the direct effects of treatment on growth, as well as the indirect effects, expressed through treatment‐driven changes in relationships between tree growth and the environment. Specifically, we asked the following questions: (Q1) How do tree‐level radial growth rates compare between treated and untreated units, and how has individual tree growth changed relative to pre‐treatment baseline conditions? We expected that tree growth would be higher in treated units and that most trees would respond positively to treatment, whereas tree growth in untreated areas would decline over time due to recent warm and dry conditions. (Q2) How does treatment alter the relationships between growth and other biophysical factors? We expected that treatment would have a direct, positive influence on tree growth across sites, but that they would have a comparatively greater effect at wetter sites, where competition is more limiting. We also expected that small‐ to intermediate‐sized trees would respond more positively to treatment, that local stand conditions (e.g., basal area) would create variable patterns of growth within both treated and untreated areas, and that treatment would reduce the sensitivity of tree growth to interannual climate variation.

## METHODS

### Study sites and experimental design

Our study sites are part of the Long‐Term Ecological Assessment and Restoration Network (LEARN), a system of permanent experimental units located in the southwestern United States (Table 1, Figure 1). LEARN began in the late 1990s, and each site features the same randomized, replicated experimental design that includes prescribed fire and a locally specific thinning prescription based on pre‐industrial (i.e., mid to late 1800s) patterns of forest structure, tree species composition, and spatial arrangement (Moore et al., 1999; Stoddard et al., 2021; Tuten et al., 2015). The present study includes five sites in Arizona, USA, that were thinned >10 years before field surveys in 2019. Within each site, one to four replicated blocks (*n* = 14 blocks across sites; each ca. 30 ha) span local gradients of soil type and productivity. All blocks contain a paired treated unit and untreated unit, each with twenty 400 m^2^ (11.28 m radius) monitoring plots located on a 60‐m systematic grid (*n* = 559 plots across sites). Thinning within treatment units followed a single‐tree selection prescription focused on approximating pre‐industrial stand conditions (see Tuten et al., 2015). All but one treatment unit (i.e., CF—Block 1) received at least one entry with prescribed fire after thinning was completed.

**TABLE 1:** Total sample sizes (in numbers), the percentage of pre‐treatment basal area and trees removed within treated units, soil parent material, and management agency at each of five experimental sites in Arizona, USA.

Site	No. plots		No. trees		No. growth rings^a^		Basal area removed (%)	Trees removed (%)	Soil parent material	Management agency^b^
	U	T	U	T	U	T				
Apache‐Sitgreaves (AS)	34	26	91	42	3927	487	62.1	85.3	Volcanic	USFS
Centennial Forest (CF)	51	45	88	66	4237	945	43.6	83.5	Basalt	AZ State
Fort Valley (FV)	45	30	92	52	3825	1040	53.2	84.1	Basalt	USFS
Grandview (GV)	15	10	41	14	1586	266	66.1	89.7	Sandstone/limestone	USFS
Mt. Trumbull (MT)	62	41	110	74	4763	1412	59.0	78.2	Lava/cinder/basalt	NPS/BLM
All sites	207	152	422	248	18,338	4150	53.8	82.5		

*Note*: Untreated (U) and treated (T) samples are defined as those that were withheld from treatment or experienced restoration treatment, respectively.

^a^ Growth rings within treated units were considered to have an untreated status here and in subsequent analyses until after thinning was implemented on a plot. All rings from trees within untreated units had an untreated status throughout the study period.

^b^ USFS, USDA Forest Service; AZ State, Arizona Department of Forestry and Fire Management; NPS, DOI National Park Service; BLM, DOI Bureau of Land Management.

Ponderosa pine was the most abundant tree species (67%–100% of total tree basal area) at all five sites in 2019. Additional tree species included Gambel oak (*Quercus gambelii* Nutt.), New Mexico locust (*Robinia neomexicana* A. Gray), three juniper species (*Juniperus monosperma* (Engelm.) Sarg.; *Juniperus osteoperma* (Torr.) Little; *Juniperus deppeana* Steud.), two‐needle pinyon pine (*Pinus edulis* Engelm.), and/or southwestern white pine (*Pinus strobiformis* Engelm.). Across sites, mean annual temperatures (i.e., 1991–2020) span 7.4–10.2°C, and mean annual precipitation spans 395–679 mm year^−1^ (Thornton et al., 2021). Overall, these sites encompass 50% of the distribution of temperature and 83% of the distribution of precipitation for Rocky Mountain ponderosa pine (*P. ponderosa* var. *scopulorum*) (Little, 1971; Shinneman et al., 2016; Wilson et al., 2013) (Figure 1a,b).

### Data—Response variable

To quantify tree radial growth rates, we collected increment cores from a stratified random sample of all live ponderosa pine present on our sites in 2019 (Table 1). We developed sampling strata across blocks, treatments, plots, and tree sizes. One increment core was collected from each selected tree at 40 cm above ground level, perpendicular to the slope to reduce the effects of compression and tension wood on measured growth rates. We mounted and prepared increment cores using progressively finer grit sandpaper until ring margins were visible (Speer, 2010). Each core was visually crossdated against preexisting chronologies from each site, and a second analyst confirmed the correct dating of each sample. We then recorded ring widths using a measuring stage (Velmex, Bloomfield, NY) and Measure J2X software (VoorTech Consulting, Holderness, NH) and verified crossdating using COFECHA (Holmes, 1983). Mean inter‐series correlations (Pearson's *r*) ranged from 0.65 to 0.82 across sites.

For each year of tree growth, we calculated BAI, which represents the annual increase in cross‐sectional area of a tree at coring height and is a strong proxy for carbon accumulation (Babst et al., 2014). Calculations of BAI require both annual ring widths and the bark‐free diameter of a tree at coring height. Therefore, we used stem taper and bark thickness equations developed for ponderosa pine in the southwestern United States (Rodman et al., 2017, 2021) to predict the bark‐free diameter of each tree in 2019 from field‐measured diameter at breast height (i.e., 1.37 m above ground) in the same year. We then used these diameter estimates and measured ring widths as inputs to the “bai.out” function in the dplR package (Bunn, 2010; v. 1.7.4) in R (R Core Team, 2022; v. 4.2.2) to calculate BAI (in square millimeters per year). We restricted data in our analysis to rings formed from 1985 to 2018 based on the availability of model covariates, yielding 22,488 annual BAI values from 670 trees (Table 1). We applied a power transformation following the approach of Cook and Peters (1997), modified to accommodate BAI data, to stabilize variance and meet the distributional assumptions of our statistical model. The final response variable was annual BAI (in square millimeters per year)^0.24^ (hereafter “power‐transformed BAI”).

### Data—Predictors of radial growth

As predictors of annual BAI, we developed covariates that represented the local competitive environment, individual tree size, interannual climate variability, average site conditions, and treatment. Brief descriptions of these covariates are provided in Table 2, and more detailed descriptions are provided in Appendix S1: Section S1. We described treatment effects using a binary variable (hereafter “treatment status”), which specified whether a given growth ring was formed before (i.e., untreated) or after (i.e., treated) thinning had occurred within a block and treatment unit. Thus, trees in untreated units had an “untreated” status in all years, whereas trees in treated units had an “untreated” status until local treatment implementation occurred, and then a “treated” status for all subsequent years.

**TABLE 2:** Predictors of ponderosa pine basal area increment (BAI) at five experimental sites across Arizona, USA; their rationale for inclusion; and the spatial and temporal scales at which they were summarized.

Variable	Methods/rationale for inclusion	Spatial/temporal resolution
Basal area within treatment (BA)	Obtained from field surveys at each plot and converted to annual values using linear interpolation between surveys with constant extrapolation outside of survey years. An indicator of local competition, within a treatment.	Episodic (2–4 surveys per plot, completed in specific years). Converted to annual (year of growth ring). Plot‐scale (0.04 ha).
dbh	Reconstructed from 2019 field data and cumulative tree ring widths from prior years. Tree size affects the environmental niche of an individual. Square‐root‐transformed to linearize the relationship between dbh and growth.	Annual (year prior to growth ring). Tree‐scale.
Available soil water (ASW)	The amount of soil moisture in the top 1.5 m of the soil profile (held at >−3.0 MPa) using a daily soil water balance model. An indicator of spatial and temporal variation in belowground moisture availability within a site.	Seasonal (mean daily values within each of five seasons) for year of growth ring and previous 4 years. Plot‐scale (0.04 ha).
Vapor pressure deficit (VPD)	Calculated as the difference between mean vapor pressure and saturation vapor pressure on a given day. An indicator of spatial and temporal variation in atmospheric evaporative demand within a site.	Seasonal (mean daily values within each of five seasons) for year of growth ring and previous 4 years. Block‐scale (20 ha).
Site	The study site on which a tree was located. Overall growth and response to treatment may vary across environmental gradients.	Time‐invariant. Site‐scale.
Treatment	Whether a treatment unit had been thinned prior to the year of growth. Forest thinning was the first treatment at each site and may influence growth directly and indirectly.	Annual (year of growth ring). Treatment unit‐scale (10 ha).
Prior BAI	Tree growth is an autocorrelated process, and prior BAI can be used as an autoregressive term to help account for this.	Annual (year prior to growth ring). Tree‐scale.

### Statistical modeling

To quantify drivers of growth, we implemented a hierarchical linear regression to model power‐transformed BAI for each tree and year as a function of covariates listed in Table 2. Radial growth is influenced by climatic conditions in prior years due to their effects on longer term tree physiological processes (Anderegg et al., 2015; Peltier & Ogle, 2019). Therefore, we incorporated antecedent effects of above‐ (vapor pressure deficit; VPD) and belowground moisture stress (available soil water; ASW) using the stochastic antecedent modeling (SAM) framework (Ogle et al., 2015; Peltier et al., 2018). SAM treats individual climate terms as a weighted average of conditions across multiple time steps, quantifying their relative importance in the overall climate effect; it has typically been used in southwestern US forests to describe the importance of climate during the year of growth and the preceding 4 years (Peltier et al., 2018; Yocom et al., 2022). Our model structure included plot‐level basal area during the year of tree growth, the size (i.e., square‐root‐transformed dbh) of the tree in the preceding year, and antecedent terms for ASW and VPD (Table 2; Appendix S1: Equation S2). Because trees of different sizes might respond differently to climate, we also included two‐way interactions for ASW × dbh and VPD × dbh. Likewise, because the two climate variables might interact to affect tree growth, we included an ASW × VPD interaction.

Tree‐ring data have inherent temporal autocorrelation (Klesse et al., 2023). Therefore, we included power‐transformed BAI from the preceding year as an autoregressive term that was estimated jointly with antecedent climate effects. We used intercept terms for each treatment and site combination to assess the direct effects of treatment on baseline growth (Appendix S1: Equation S3). The tree‐level intercepts were modeled hierarchically, which allowed us to account for the nested spatial structure (i.e., trees were nested within experimental blocks, nested within sites) and the temporal structure of the data (i.e., a tree within a treatment unit may have either a treated or untreated status, depending on the year) while testing for differences in treatment effects across environmental gradients. We hierarchically centered these intercept terms, incorporating spatial and temporal structures, to ensure identifiability of the intercepts and the random effects (Ogle & Barber, 2020). To understand how treatment might alter the relationship between BAI and other covariates, we allowed the effects of basal area, dbh, ASW, VPD, and all two‐way interactions to vary by treatment status. We assumed that important seasons and the duration of climate effects (i.e., antecedent weights) would be similar for rings with treated and untreated status, but that the overall effects of climate would differ. We implemented our SAM model within a Bayesian framework, and we used relatively non‐informative priors for all stochastic parameters (e.g., regression coefficients, antecedent weights, variance terms). Specifications of the likelihood function and model components are presented in Appendix S1: Section S2.

We fit the above hierarchical model to our data using JAGS software (v. 4.3.0) and the jagsUI package in R (Kellner, 2021; v. 1.5.2) for model development. Based on the Raftery–Lewis diagnostic (Raftery & Lewis, 1992), we ran our final model for 20,000 iterations for each of three chains with a 1000‐iteration burn‐in period. We then used trace plots (Curtis, 2018) and the Gelman–Rubin statistic (i.e., all $\hat{t}$ values were <1.05; Gelman & Rubin, 1992) to assess model convergence. After burn‐in, we computed the posterior median and 95% credible intervals based on the sampled values for each parameter. To evaluate whether treatment influenced the relationships between each covariate and BAI, we calculated pairwise differences between treatment‐specific covariate effects and determined whether the 95% credible interval of these differences overlapped 0. We assessed model goodness of fit using the coefficient of determination (i.e., Bayesian *R* ^2^; Gelman et al., 2019) and root‐mean‐square error (RMSE) obtained from a comparison of observed versus predicted values of power‐transformed BAI. Because antecedent terms (i.e., ASW and VPD) had a distribution that differed from that of the scaled and centered variables, we also calculated effect sizes—the change in the response for one SD of change in the covariate—which can be more directly compared with other covariate effects. To evaluate changes in treatment effects over time and the effects of treatment intensity, we extracted model residuals for treated trees and compared them with time since thinning, time since burning, and the percentage of plot‐level basal area removed during treatment (Appendix S1: Section S3).

## RESULTS

**FIGURE 2:**
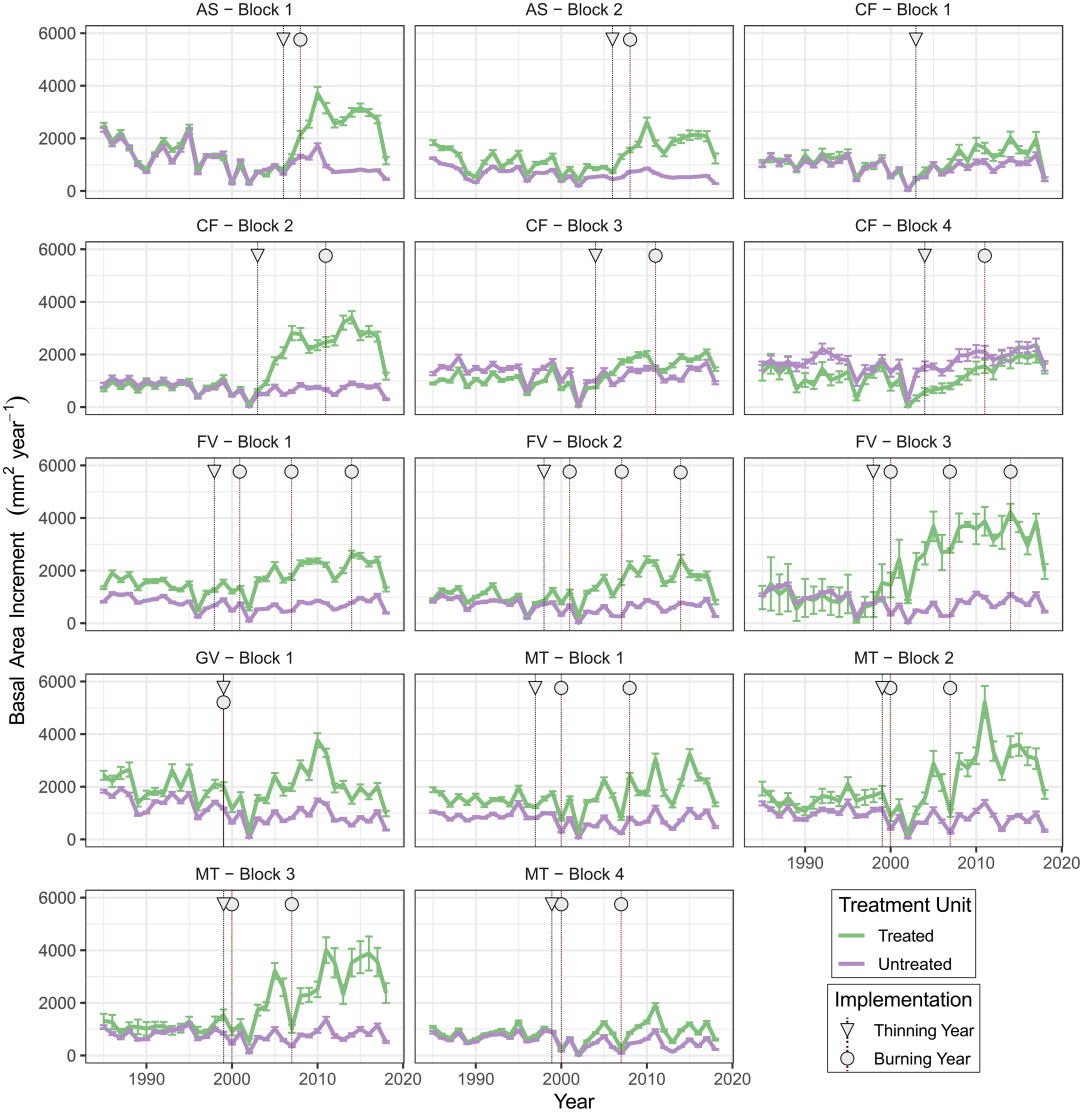
Average annual basal area increment (BAI) of sampled ponderosa pine trees (*n* = 670) from 1985 to 2018 across experimental blocks spanning five sites in Arizona, USA. Lines show the mean annual BAI for all sampled trees within a block and treatment unit, while error bars give 95% CIs (±1.96 × SE of the mean). Treatments (i.e., forest thinning and prescribed burning) were implemented in different years at each block, with lines and shapes identifying the years of treatment implementation. Only years after initial thinning were considered “treated” in subsequent statistical models. AS, Apache‐Sitgreaves; CF, Centennial Forest; FV, Fort Valley; GV, Grandview, MT, Mt. Trumbull.

Overall, tree‐level radial growth (i.e., BAI) increased due to thinning and burning treatments. Before treatment, there were relatively minor differences in mean annual BAI between trees in treated (1153 mm^2^ year^−1^) and untreated units (983 mm^2^ year^−1^). However, after treatment implementation, mean BAI was 133.1% higher in treated units (1783 mm^2^ year^−1^) than in untreated units (765 mm^2^ year^−1^). Despite drier climatic conditions over time, 73.8% of individual trees in treated units showed increases in BAI following treatment implementation, for an average increase of 85.6% relative to pre‐treatment baselines. In contrast, 77.1% of trees in untreated units showed declines in BAI during the post‐treatment period, for an average decline of 17.2%. BAI within treated areas generally responded more strongly to the timing of thinning than the timing of burning (Figure 2). Though thinning and burning had generally positive effects on annual BAI, there was notable variation in how trees in different experimental blocks responded to treatment (Figure 2).

**FIGURE 3:**
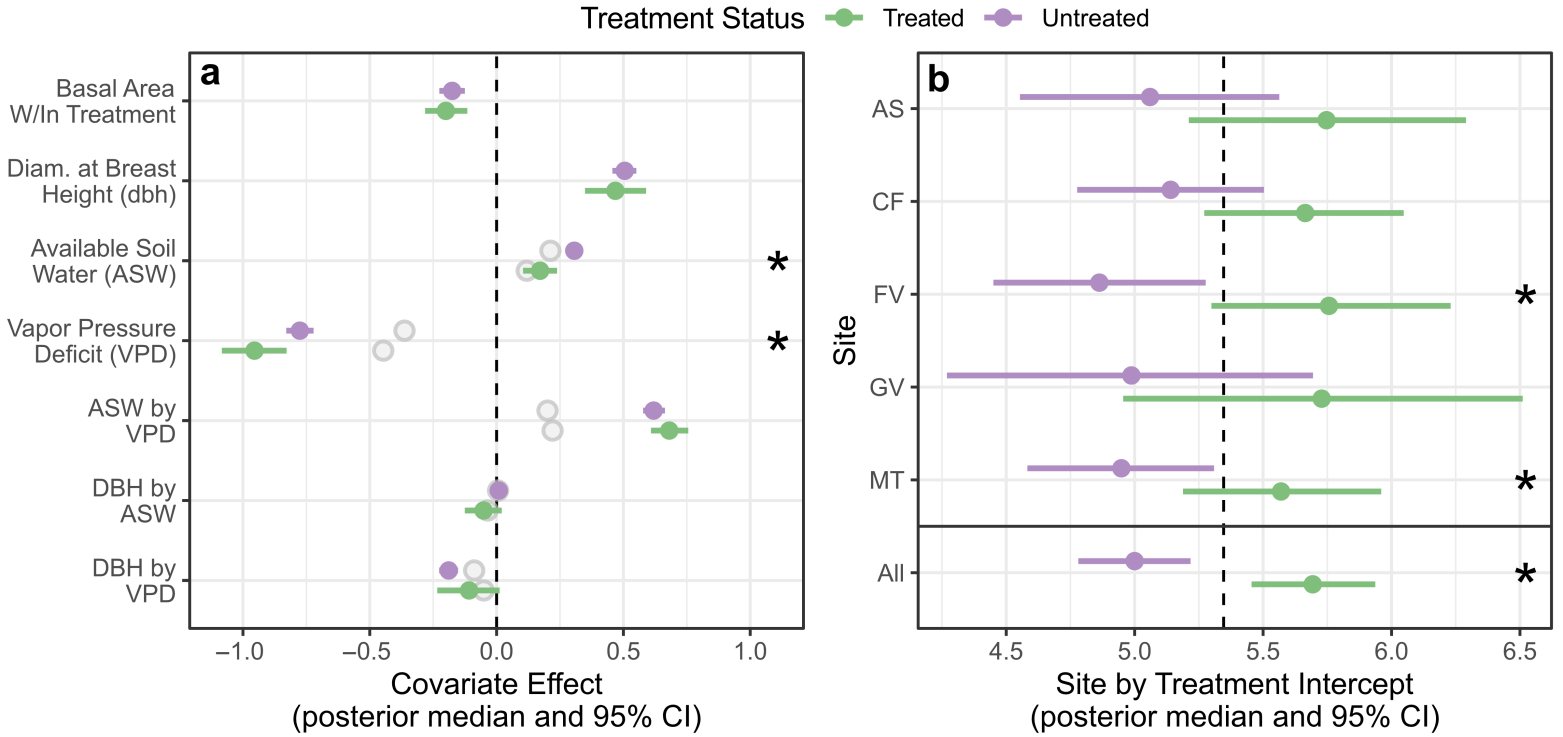
Effects of thinning and burning treatments, environmental conditions, and tree size in the hierarchical linear model of ponderosa pine basal area increment (BAI) on five experimental sites throughout Arizona, USA. Posterior medians (points) and 95% credible intervals (lines) (a) show the effects of each covariate on power‐transformed annual BAI in treated (green) and untreated (purple) trees. For antecedent climate terms (available soil water and vapor pressure deficit) and interactions, gray circles show effect sizes which can be more directly compared with the effects of basal area and diameter at breast height. Panel (b) shows site by treatment intercepts which describe average growth (i.e., power‐transformed BAI) in each site and treatment combination given the mean values of other covariates, and the vertical dashed line shows average growth across all sites and treatments. Pairwise comparisons with 95% credible intervals that do not overlap 0 (i.e., a meaningful treatment effect) are indicated with an asterisk. Site codes in (b) are as follows: Apache‐Sitgreaves (AS), Centennial Forest (CF), FV (Fort Valley), GV (Grandview), and MT (Mt. Trumbull).

**FIGURE 4:**
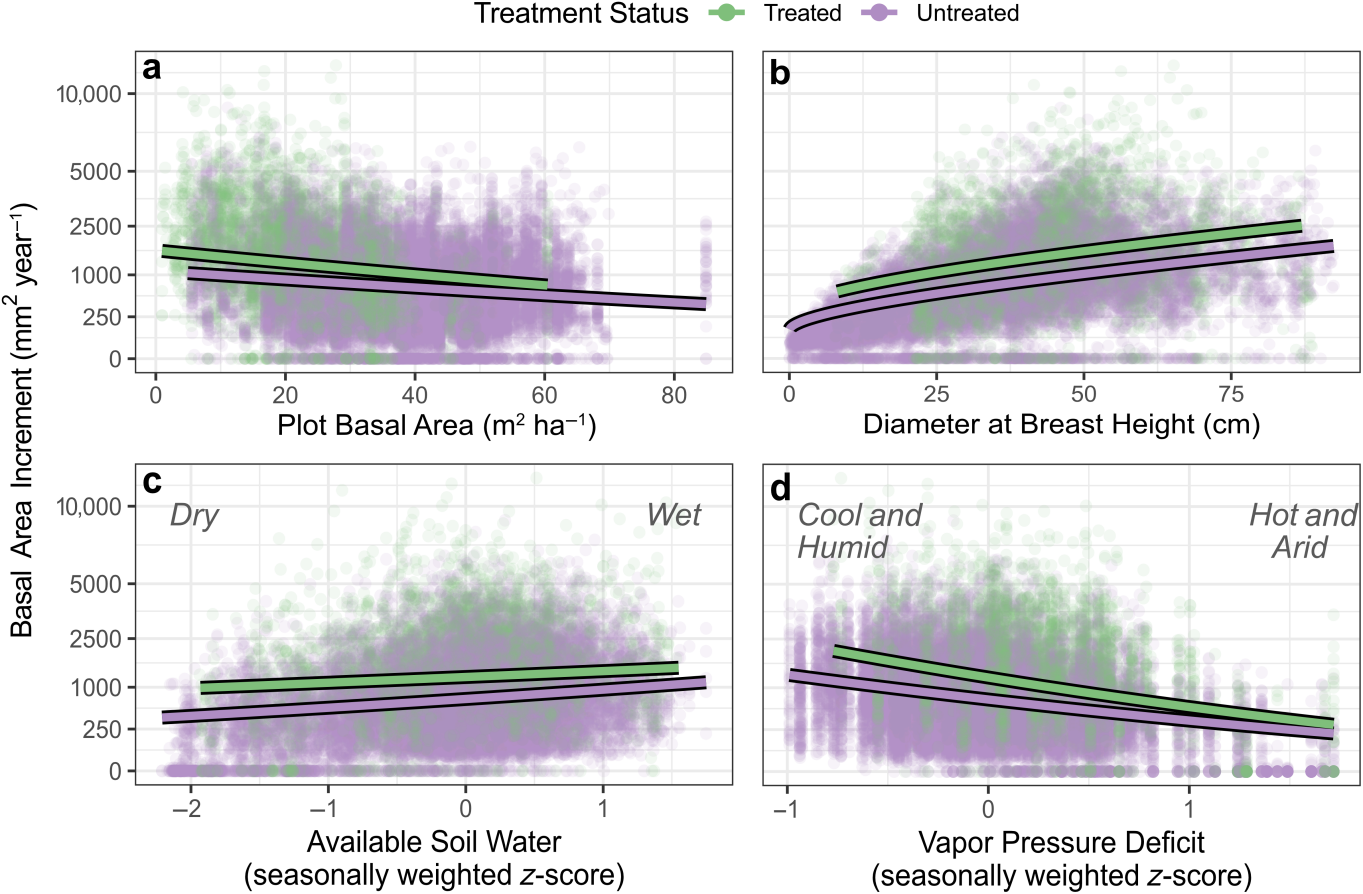
Effects of two‐way interactions between individual covariates and thinning and burning treatment in the hierarchical linear model of ponderosa pine basal area increment across five experimental sites in Arizona, USA. Treated (green) and untreated (purple) lines show model‐predicted values across the range of each covariate (using posterior median covariate effects and the mean values of other covariates), and points show observed data. Note that the *y*‐axis is on a square‐root‐transformed scale to better illustrate the range of the observed data.

**FIGURE 5:**
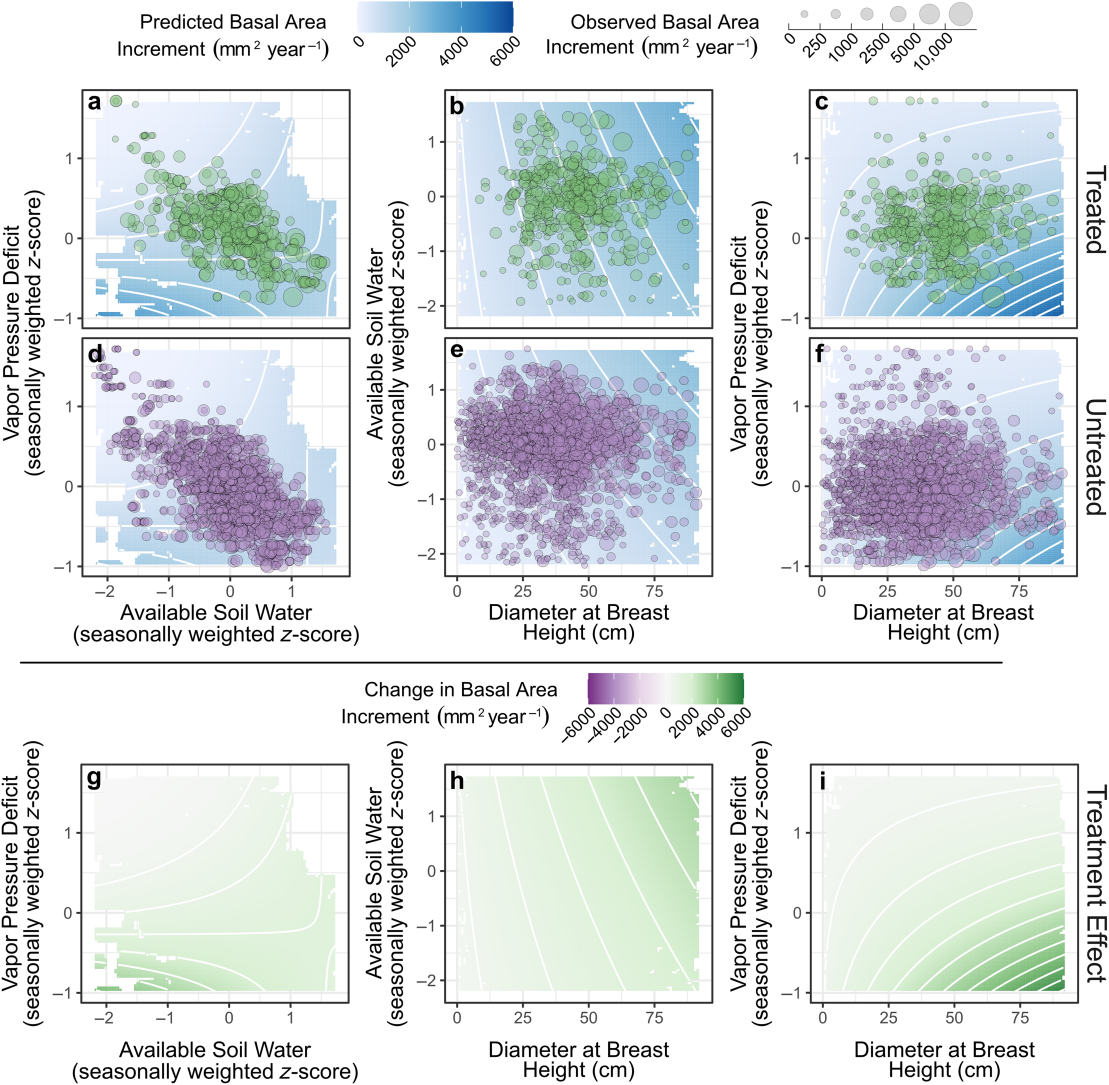
Effects of three‐way interactions between covariate pairs and thinning and burning treatment (a–f) in the hierarchical linear model of ponderosa pine basal area increment (BAI) across five experimental sites in Arizona, USA. In (a–f), background shading (white to blue) shows model‐predicted values across the ranges of each pair of covariates (using posterior median covariate effects and the mean values of all other covariates) in treated (top) and untreated (bottom) groups. Contours are at intervals of 500 mm^2^ year^−1^ to highlight patterns in predicted BAI values, and background shading is removed in areas without observed data. Green (treated) and purple (untreated) points show a 10% subset of the observed data, and point size is scaled by annual BAI. In (g–i), background shading shows the difference between predictions of growth in treated (a–c) and untreated (d–f) groups, with higher values showing a greater difference between groups.

Our hierarchical model helped to explain variation in treatment responses, demonstrating that thinning and burning directly increased BAI and influenced climate–growth relationships (Figure 3). For most covariate effects, 95% credible intervals did not overlap 0, indicating meaningful relationships with BAI (Figure 3a; Appendix S1: Table S2). Plot‐level basal area was negatively related to BAI, with relatively similar effects in each treatment (Figures 3a and 4a). Likewise, larger trees (i.e., greater dbh) had higher BAIs than small trees, and this relationship was unaffected by treatment (Figures 3a and 4b). Soil moisture (i.e., high antecedent ASW) was positively related to BAI but became less important in treated areas (Figures 3a and 4c). In contrast, VPD was negatively associated with BAI and became more important in treated areas (Figures 3a and 4d). ASW and VPD interacted, such that combinations of high values of VPD and low values of ASW were more limiting than each factor alone (Figures 3a and 5a,d), and this interaction did not vary by treatment. Interactions between dbh and interannual climate variables suggested that trees of different sizes responded similarly to ASW, although larger trees were generally more sensitive to VPD (Figures 3a and 5b,c,e,f). Treatment increased BAI (Figure 3b), but this effect was not stronger on wet sites. Thus, thinning and burning had a generally positive effect on BAI, and these effects were consistent across environmental gradients (Figures 1 and 3b). Treatment effects persisted throughout the ca. 20‐year study period, and treatment intensity did not explain additional variation that was unaccounted for by other model covariates (Appendix S1: Figure S2).

**FIGURE 6:**
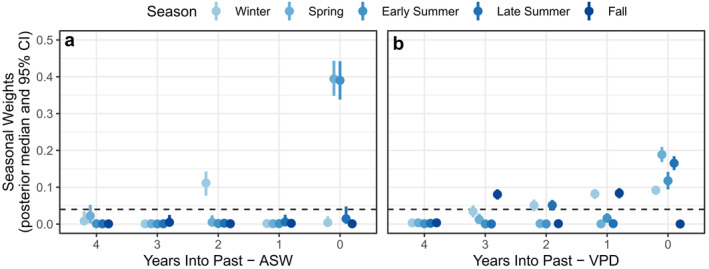
Effects of antecedent climate on annual ponderosa pine basal area increment (BAI) at five experimental sites throughout Arizona, USA. Seasonal weights (*y*‐axes) give the proportion of the overall effect of a climate variable on BAI (shown in Figure 2a) during the year of growth and the preceding 4 years. Dashed lines represent a null hypothesis of uniform effects across all seasons and years. Seasons were defined a priori based on the timing of photosynthesis and radial growth as follows: winter (prior December to current February), spring (March and April), early summer (May and June), late summer (July to September), and fall (October and November). ASW, available soil water; VPD, vapor pressure deficit.

Antecedent climate effects illustrated that ASW and VPD influenced BAI over different timescales. For example, soil moisture during the year of growth accounted for 80.4% of the total effects of ASW, with only 19.6% of seasonal weights assigned to prior years (Figure 6a). The strongest relationships between soil moisture and BAI were in the spring and early summer months (i.e., March to June) during the year of growth. In contrast, the relationship between BAI and atmospheric aridity exhibited greater lagged effects, with conditions during the year of growth accounting for just 56.4% of the overall effects of VPD (Figure 6b). The strongest relationships between VPD and BAI were in spring and summer months during the year of growth and winter or fall months in prior years. The hierarchical model performed well, with a high coefficient of determination (Bayesian *R* ^2^ = 0.75), a low root‐mean‐square‐error (RMSE = 1.11; 22.6% of the response mean), and predicted values that closely aligned with the 1:1 line (Appendix S1: Figure S1).

## DISCUSSION

We leveraged data from a long‐running (i.e., >20 years) network of experimental sites in the southwestern United States (Moore et al., 1999; Springer et al., 2023; Stoddard et al., 2021) to investigate the effects of thinning and burning treatments on ponderosa pine radial growth rates and better understand how these effects varied across environmental gradients. We focused specifically on radial growth because it is a holistic indicator of individual tree vigor, carbon accumulation, and defense (Babst et al., 2014; Gonzalez et al., 2023; Kane & Kolb, 2010). Our experiment began at the onset of an intense drought period (Williams et al., 2022), thereby providing a unique opportunity to evaluate the effectiveness of treatments at mitigating drought stress effects under extreme climatic conditions, an analog for the warmer, drier conditions expected in upcoming decades (Thorne et al., 2018; Triepke et al., 2019). We are aware of only one other empirical study in the western United States that has examined the effects of thinning and burning treatments across the climatic range of a tree species (i.e., Young et al., 2023). However, that study did not employ formal experimental treatments and occurred in a wet (i.e., 871–1441 mm of annual precipitation), Mediterranean climate, in contrast to our controlled experimental design and drier (i.e., 395–679 mm of annual precipitation), bimodal winter‐summer precipitation regime. Our results demonstrated that thinning and burning enhanced tree radial growth rates throughout the southwestern United States and altered climatic constraints on growth.

### Thinning and burning treatments enhance tree radial growth across biophysical gradients

Average tree BAI increased due to thinning and burning treatments throughout our study area where trees in treated units grew 133.1% faster than untreated units and 85.6% faster than their pre‐treatment baselines. These findings align with prior studies showing the positive effect of thinning on tree radial growth and drought resistance for ponderosa pine and other species (D'Amato et al., 2013; Hood et al., 2024; Sohn et al., 2016; Zald et al., 2022). However, contrary to prior research (Gleason et al., 2017) and established theory (Bertness & Callaway, 1994), treatment effects were consistent across broad‐scale environmental gradients. Indeed, one other study evaluated treatment effects on ponderosa pine across climate gradients in California and showed comparatively greater effects in wet areas (Young et al., 2023), which contrasts with our results. Differences in these findings may be due to the relatively open post‐treatment conditions at our sites (Table 1) or differences in sampling schemes; tree densities, stand basal areas, and sampled tree diameters were substantially lower in the present study than the treatment units of Young et al. (2023). Likewise, most of our sites experienced thinning treatments followed by prescribed fire, whereas the sites of Young et al. (2023) primarily included thin‐only or burn‐only treatments. Prior to treatment, it is likely that most of our sites were near their carrying capacity of plant biomass (Bond & Keeley, 2005; Jaquette et al., 2021). Thus, we infer that an ~80% reduction in density and 50% reduction in basal area at each site provided a similar treatment effect across sites (Table 1). Overall, our results suggest that thinning and burning treatments—which reduced stand densities to near pre‐industrial conditions and reintroduced fire—had a direct, positive, and enduring effect on tree‐level radial growth during a period of extreme drought (Williams et al., 2022), an effect that was relatively consistent across broad‐scale environmental gradients of precipitation, temperature, and soil type.

### Climate influences tree growth and treatments Alter climatic constraints

Antecedent climate effects included in our models improved the understanding of how the above‐ (VPD) and belowground (ASW) components of moisture stress influence tree growth over multi‐year timescales. We found that ASW in the spring and early summer months (i.e., March to June) during the year of growth was most influential on tree BAI, without strong lag effects from preceding years. Likewise, the effects of VPD were apparent during the spring and summer months in the year of growth (i.e., March to September), but VPD during prior years also had important lagged effects. Stable isotope measurements illustrate that winter precipitation and spring soil moisture are a dominant source of water for ponderosa pine in the Southwest (Bailey et al., 2023; Kerhoulas et al., 2013). High temperatures and evaporative demand during the warm summer months are also known to limit tree growth for this species (Peltier et al., 2018). Thus, consistent with prior research (Andrews et al., 2020; Williams et al., 2012), our results demonstrate that winter precipitation and summer drought stress are key climatic constraints during the year of tree growth. Together, these factors can create variation in turgor pressure, which is a primary driver of radial growth (Peters et al., 2021). Moreover, we found that lagged effects of VPD in the fall and winter months (i.e., October to February) of preceding years had an important effect on radial growth, which may relate to hydraulic damage or the production, storage, and allocation of nonstructural carbohydrates (Peltier et al., 2022; Trugman et al., 2018). For ponderosa pine in the Southwest, new root growth, needle development and elongation, and total photosynthetic production also peak in the late summer to fall (i.e., August to October) (Gaylord et al., 2007). Thus, prior fall or winter VPD may influence tree physiological processes through multiple pathways—hydraulic injury, nonstructural carbohydrate availability, and recent growth of tissues supporting water uptake and photosynthesis—providing a mechanism by which lagged climatic effects might influence radial growth in ponderosa pine.

Thinning and burning treatments also shifted tree responses to interannual climate variability in our study area, decreasing the effects of ASW and increasing the effects of VPD. In our study area, tree removal commonly increases soil moisture availability in deeper soil horizons, which are important for both saplings and large trees (Belmonte et al., 2022; Kerhoulas et al., 2023). For example, after treatment implementation at our sites, modeled ASW was ~6% higher in treated areas during the spring and early summer seasons, even after accounting for post‐treatment increases in understory herbaceous cover (e.g., Springer et al., 2023) and evapotranspiration by remaining trees (Appendix S1: Figure S4). These treatment‐driven increases in ASW may decouple relationships between tree growth and soil moisture (Andrews et al., 2020). In contrast, forest thinning can increase canopy temperatures (Sankey & Tatum, 2022) and subcanopy wind speeds and turbulence (Russell et al., 2018). Such changes are also likely to increase evaporative demand experienced at the stomata. Consequently, our findings and those of other studies suggest that treatment alters the climatic constraints on tree growth, reducing the effects of belowground moisture availability and increasing the effects of aboveground evaporative demand. So far, treatment‐driven increases in soil moisture have offset any potential increases in evaporative demand, enhancing the ability of trees to maintain transpiration and growth under extreme drought (this study; Sankey & Tatum, 2022). However, such treatment effects may wane over time. Climate models consistently project increases in temperature and evaporative demand throughout the Southwest, with greater uncertainty in projections of precipitation and soil moisture (Bradford et al., 2020; McDowell et al., 2016).

### Tree size and forest basal area promote variable growth patterns

Tree size (i.e., dbh) had important effects on both climate sensitivity and BAI. For example, we found that while trees of different sizes had relatively consistent relationships with soil moisture, BAI in larger trees was especially sensitive to atmospheric evaporative demand. These relationships between tree size and climate sensitivity were unaffected by treatment. Larger trees can be highly vulnerable to drought events because of the greater hydraulic conductivity needed for water transport to the upper canopy and because of the higher evaporative demand experienced by exposed crowns (Bennett et al., 2015; Trugman et al., 2021). Such individuals also play a disproportionate role in many ecosystem processes (Lindenmayer et al., 2012). Indeed, as shown in the present study through positive relationships between BAI and tree size, larger individuals often have greater rates of accumulation of woody biomass. Although large trees are rare in many landscapes (Kane et al., 2023), a range of possible approaches exist to maintaining or increasing their prevalence through management (Baker, 2021; Kolb et al., 2007). Where feasible, restoration treatments, which often select for the retention of larger trees and enhance tree‐level growth, are likely to increase the number of large trees on the landscape and promote old‐growth structural conditions over time (Case et al., 2023). We also found that thinning and burning consistently increased BAI across tree sizes, contrary to prior research suggesting that small‐ to intermediate‐sized trees can disproportionately benefit from restoration treatments (Fulé et al., 2022; Skov et al., 2005). Such differences might be attributed to variations in analytical methods among studies. For example, we surveyed a wide range of tree sizes and used linear models. Thus, if intermediate‐sized trees are those that respond most strongly to treatment (Roccaforte et al., 2024; Young et al., 2023), this nonlinear pattern would not be fully captured in our modeling approach. Overall, restoration treatments did not alter interactions between tree size and interannual climate variables and consistently enhanced BAI across tree sizes.

Variation in local tree basal area promoted variable patterns of tree BAI in both treated and untreated areas. Reconstructions of forest structure in ponderosa pine forests of the southwestern United States suggest that basal areas ranged from at least 5.9 to 28.5 m^2^ ha^−1^ at the stand scale prior to fire regime disruption in the late 1800s (Reynolds et al., 2013), but there was substantial local and subregional variability in forest density (Rodman et al., 2017; Williams & Baker, 2012). Even under active fire regimes, patches of high‐density forests, interspersed with low‐density savannahs and meadows, were likely an important component of the landscape that provided critical habitat for species such as spotted owl (*Strix occidentalis*) and tassel‐eared squirrel (*Sciurus aberti*) (Dodd et al., 2006; Jones et al., 2020). Fine‐scale variation in spatial patterns—including tree groups of a range of sizes and densities, isolated individuals, and openings—is also an essential component of many dry forests throughout the western United States (Chamberlain et al., 2023; Larson & Churchill, 2012). Such patterns are likely to influence various ecological processes (Tuten et al., 2015). For example, local variation in tree growth rates, driven by fine‐scale inter‐ or intraspecific interactions, might help to reduce landscape‐scale susceptibility to bark beetle outbreaks (Seidl et al., 2016). Local forest density and basal area also influence reproductive processes such as cone production and tree establishment (Flathers et al., 2016; Rodman et al., 2021). Overall, denser stands store more carbon and accumulate carbon more quickly than low‐density stands (Andrews et al., 2020; Bradford et al., 2021). Thus, while stands with low basal areas and evenly spaced trees are likely to have the greatest rates of tree‐level growth, incorporating variable densities and spatial patterns of trees into silvicultural prescriptions is consistent with the evolutionary history of these forests and is likely to meet multiple management objectives.

## CONCLUSION

While we primarily focused on the effects of thinning and burning treatments on tree growth, such treatments can also influence wildfire behavior (Cansler et al., 2022; Davis et al., 2024; Kalies & Yocom Kent, 2016), tree defense (e.g., resin duct production; Hood et al., 2015; Wallin et al., 2008), herbaceous plant cover and richness (Demarest et al., 2023; Hood et al., 2024; Springer et al., 2023), and avian diversity (Latif et al., 2020). Still, the financial costs of thinning and burning treatments can be considerable, particularly in remote areas with steep terrain (Chang et al., 2023), and they are unlikely to be implemented in all dry forests. Indeed, such strategies are just one of many tools that can be utilized by managers in a larger framework to resist, accept, or direct ecosystem transformation (Schuurman et al., 2022). Drought events are increasing globally (Hartmann et al., 2022; Moss et al., 2024), and management activities such as forest thinning and burning may help to promote near‐term drought resistance to allow for adaptation and persistence of critical populations or habitat features. Over upcoming decades, new approaches, such as accepting or directing ecosystem transformations, may be needed given widespread projected increases in atmospheric evaporative demand across western US dry forests.

## AUTHOR CONTRIBUTIONS

Kyle C. Rodman, David W. Huffman, and Michael T. Stoddard conceived the ideas. Peter Z. Fulé, David W. Huffman, Rory J. Pedersen, Michael T. Stoddard, and Amy E. M. Waltz collected the field data. Donald P. Normandin, Rory J. Pedersen, and Michael T. Stoddard conducted laboratory work. John B. Bradford, Thomas E. Kolb, and Daniel R. Schlaepfer processed climate data and/or helped select variables. Kyle C. Rodman, Alicia M. Formanack, Ana T. Miller‐ter Kuile, and Kiona Ogle designed statistical methodology and analyzed the data. Kyle C. Rodman led the writing of the manuscript. All authors contributed critically to drafts and gave final approval for publication. In the first page, authors 2 to 13 are ordered alphabetically, and not ranked in terms of contribution.

## CONFLICT OF INTEREST STATEMENT

The authors declare no conflicts of interest.

## Supporting information


Appendix S1:

## Data Availability

Data, analytical code, and statistical model outputs (Rodman et al., 2024) are available in Zenodo at https://doi.org/10.5281/zenodo.13952418.
