# Supplementary material for: Restoration treatments enhance tree growth and alter climatic constraints during extreme drought
Source: Ecol Appl. 2024 Dec 3;35(1):e3072. doi: 10.1002/eap.3072 (PMC11726003; doi:10.1002/eap.3072)
Supplement: Supplementary file 1 — Appendix S1: [file EAP-35-e3072-s001.pdf]

Appendix S1 for: Restoration treatments enhance tree growth and alter climatic constraints during extreme drought

Authors: Kyle C. Rodman, John B. Bradford, Alicia M. Formanack, Peter Z. Fulé, David W. Huffman, Thomas E. Kolb, Ana T. Miller-ter Kuile, Donald P. Normandin, Kiona Ogle, Rory J. Pedersen, Daniel R. Schlaepfer, Michael T. Stoddard, Amy E.M. Waltz

Journal: *Ecological Applications*

Section S1: Developing Predictors of Tree-Level Radial Growth

As predictors of annual basal area increment (BAI), we developed covariates that represented the local competitive environment, individual tree size, interannual climate, average site conditions, and treatment (Table 2 in main text). To describe local competition, we used field-derived basal area of all live trees within the plot on which a tree was located. Individual plots were visited between two and four times throughout the study period, with one pre-treatment survey and one to three post-treatment surveys. We assumed that basal area remained constant from 1985 to the first pre-treatment survey (ca. 2000), that it declined to values of the initial post-treatment survey between the year of thinning and initial post-treatment survey, and that it changed linearly between surveys after that (i.e., linear interpolation of missing values between post-treatment surveys). In initial models, we also tested annual, Landsat-derived maps of tree canopy cover (Allred et al. 2021), which yielded qualitatively similar results to plot basal area (data not shown). While these remotely sensed canopy cover data had a finer temporal resolution, they also introduced additional uncertainty in local competitive effects; thus, we ultimately used plot-level basal area as our metric of competition. To describe tree size, we used a 2019 field inventory of tree diameter at breast height (DBH; 1.4 m above ground level). We then converted these values to an annual resolution from 1985 to 2017 by subtracting measured ring widths from 2019 tree diameter measurements and adjusting bark thickness for changes in tree size (Rodman et al. 2021). These reconstructed DBH values were strongly correlated with field-derived DBH measurements in pre-treatment census intervals (ca. 2000) at each site (r = 0.99). To linearize the relationship between tree size and growth, we square-root-transformed annual DBH values prior to analysis.

Available soil water (ASW) and vapor pressure deficit (VPD) are two key constraints on the physiological function of trees, representing the total moisture available for root uptake, and evaporative demand of the atmosphere, respectively. To calculate ASW, we used SOILWAT2, a mechanistic daily water balance model developed for dryland ecosystems and conifer forests (Schlaepfer et al. 2012, Bradford et al. 2014). We used field-derived measurements of understory vegetation (Springer et al. 2023) and forest structure at our sites (Stoddard et al. 2021), before and after treatment implementation, along with GIS-derived daily temperature and precipitation values (Thornton et al. 2021), topography (USGS, 2021), and soil data (Chaney et al. 2019) to parameterize SOILWAT2. These model inputs alter rates of interception and evapotranspiration, and modify total water storage capacity, porosity, and permeability in different portions of the soil profile. For example, reductions in tree cover after treatment enhanced available soil moisture in many treatment units, despite general increases in understory vegetation cover (Appendix S1: Figure S4). During SOILWAT2 model runs, spanning 1980 to 2021, we estimated ASW as the total soil moisture (held at > -3.0 MPa) in soil depths of 0 to 150 cm, a typical rooting depth for *P. ponderosa* (Burns and Honkala 1990). For subsequent analyses, we aggregated daily ASW values to the mean value within seasons on a given plot and year. Based on the timing of growth, photosynthesis, and past dendroclimatic relationships for the species (Gaylord et al. 2007, Andrews et al. 2020, Fulé et al. 2022), we defined seasons as follows: winter (prior December to February), spring (March and April), early summer (May and June), late summer (July to September), and fall (October and November).

To characterize VPD, we acquired mean daily vapor pressure data, as well as daily minimum and maximum temperatures from Daymet (Thornton et al. 2021). Because saturation vapor pressure (SVP) and VPD are unavailable as products via Daymet, we used improved

Magnus equations to estimate mean daily SVP from daily temperatures following Huang (2018). However, because mean daily SVP is not equal to the mean of daily minimum and maximum SVP (due to the non-linear increase in SVP with temperature), we corrected for this effect using coefficients from Williams et al. (2012). We then estimated mean daily VPD as the difference between SVP (calculated as described above) and mean Daymet-derived vapor pressure on a given day. For subsequent analyses, we aggregated daily VPD to seasonal means as above.

As a simple indicator of average abiotic conditions, we used a categorical term of “site”, which represents the site on which a tree was located (5 sites total). We assumed that this categorical variable would capture spatial variation in abiotic conditions (e.g., average temperature, moisture, soil type) experienced by plants, and help to elucidate variation in treatment effects across environmental gradients.

We described treatment effects using a binary variable (i.e., presence/absence), which specified whether a growth ring was formed after thinning had occurred on a block and treatment unit. We used completion of thinning as our criterion to define treatment because thinning was the first treatment implemented at each site and one experimental block at the CF site was never burned. Likewise, replication was too low to consider an additional level of “thinned and burned”, and thinning has had a comparatively greater and more consistent effect than burning on drought vulnerability in past research (Sala et al. 2005, Sohn et al. 2016, Steel et al. 2021) and in preliminary analyses using our data (Fig. 2 in main text). To improve model convergence and stability, we centered and scaled (i.e., converted to z-scores) all continuous predictors. For plot basal area, we scaled and centered values *within* each treatment category, to reduce relationships between basal area and the treatment variable, and focus this covariate on variations in tree basal area within a treatment. For DBH, we centered and scaled values across all sites, treatments, and

years. For ASW and VPD, we performed scaling within each site and season, such that *z*-scores represent spatial and temporal variation in climate conditions *within* a site, whereas the “site” variable characterizes overall differences in biophysical conditions *among* sites.

Section S2: Summary of Statistical Model Structure

The following provides a detailed description of the structure of our hierarchical Bayesian linear regression model of tree-level radial growth (i.e., annual basal area increment; BAI). For this model, we assumed that power-transformed BAI values (*g* = BAI^0.2436712) for ring *r*, which is associated with a particular tree and year, were normally distributed around an overall mean (μ) and variance (σ^2) as follows:

$$g_r \sim N(\mu, \sigma^2) \qquad \text{(Equation S1)}$$

Where the standard deviation, σ, was assigned a relatively non-informative uniform prior, U(0,100), and μ was modeled as a function of observed covariates.

We modeled μ as a function of antecedent available soil water (ASW) (W^{ant}), antecedent vapor pressure deficit (VPD) (V^{ant}), basal area (A) in year y, square-root-transformed tree DBH in the previous year (D^{pre}), and an autoregressive term of BAI^0.2436712 (i.e., the same power transformation applied to the response variable) in the previous year (B^{pre}). Because the two antecedent climate variables might interact to influence growth, we included the two-way interaction $W^{ant} \times V^{ant}$. Likewise, because trees of different sizes might respond differently to interannual climate, we included two-way interactions for $D \times V^{ant}$ and $D \times W^{ant}$. Because we were interested in how treatment influenced the relationships between growth and environmental factors, we allowed the covariate effects to vary by treatment t. The overall model structure was as follows:

$$\mu_r = \varepsilon_{T(r)} + \beta_{1,t(r)} W^{ant}_{y(r),p(r)} + \beta_{2,t(r)} V^{ant}_{y(r),p(r)} + \beta_{3,t(r)} A_{y(r),p(r)} + \beta_{4,t(r)} D^{pre}_r + \beta_{5,t(r)} W^{ant}_{y(r),p(r)} V^{ant}_{y(r),p(r)} + \beta_{6,t(r)} D^{pre}_r W^{ant}_{y(r),p(r)} + \beta_{7,t(r)} D^{pre}_r V^{ant}_{y(r),p(r)} + \lambda_{tr(r)} B^{pre}_r$$

(Equation S2)

Where the nested subscripting notation $T(r)$, $t(r)$, $y(r)$, $p(r)$, $b(r)$, and $tr(r)$ denote the tree-treatment indicator T, treatment t, year y, plot p, block b, and tree tr, respectively, associated with ring r. In particular, T tracks both the tree identity associated with ring r and whether or not a ring was formed after treatment had occurred. Each treatment-specific coefficient ($\beta_{j,t}$ terms, for j = 1, 2, …, 6) was assigned a relatively non-informative normal prior with mean zero and variance 1000, $N(0, 1000)$.

We provided a hierarchical prior for the intercept term, ε, such that these tree-treatment random effects were centered around block- and treatment-level intercepts ($\alpha_{b,t}$), which were centered around site- and treatment-level intercepts ($\beta_{0,s,t}$). That is, trees were nested in blocks and blocks were nested in site. The hierarchical model is as follows:

$$\varepsilon_T \sim N\left(\alpha_{b(T),t(T)}, \sigma_\varepsilon^2\right)$$
$$\alpha_{b,t} \sim N\left(\beta_{0,s(b),t}, \sigma_\alpha^2\right) \qquad \text{(Equation S3)}$$

Where the nested notation $b(T)$, $t(T)$, and $s(b)$ denotes the experimental block b, treatment t, and site b, respectively, associated with tree-treatment indicator T. All site- by treatment-level intercepts (β_0 terms) were assigned a relatively non-informative normal prior, $N(0, 1000)$, and the standard deviation terms (σ_ε and σ_α) were assigned relatively non-informative uniform priors, $U(0,100)$.

To account for temporal autocorrelation in BAI that was unrelated to tree size, antecedent climate, and their effects on tree physiological processes, we specified a hierarchical prior for the tree-level autoregressive effect (λ term in Equations S2 and S4) that accounted for trees being nested in blocks and blocks nested in sites:

$$\lambda_{tr} \sim N(\gamma_{b(tr)}, \sigma_\lambda^2)$$
$$\gamma_b \sim N(\delta_{s(b)}, \sigma_\gamma^2) \qquad \text{(Equation S4)}$$

Where the notation $b(tr)$ denotes block b associated with tree tr. The site-specific δ terms were assigned a relatively non-informative normal prior, $N(0, 1000)$, and the standard deviations (σ_λ and σ_γ) were assigned relatively non-informative uniform priors, $U(0,100)$. A one-year lag (i.e., akin to an AR(1) term), as used here, captured much of the variation in the total autoregressive effect in similar datasets (Peltier et al. 2016).

In a small number of cases (n = 7), prior BAI (i.e., power-transformed BAI_{y-1}) was missing in our data because a year (y) was not preceded by any valid ring widths (e.g., the first valid year in a series occurred after 1985). In these cases, prior BAI, B^{pre}, was imputed using the mean of all BAI estimates within a tree ($\mu(g)$) as follows:

$$B_r^{pre} \sim N(\mu_r^{pre}, \sigma_B^2)$$
$$\mu_c = \theta_B \cdot \bar{B}_{tr(r)} \qquad \text{(Equation S5)}$$

Where $\bar{B}_t$ is the mean of the observed power-transformed BAI values for tree tr. The scaling parameter, θ, and the standard deviation, σ_B, were assigned relatively non-informative uniform priors, $U(0,100)$.

Lastly, antecedent climate variables for ASW (W^{ant}) and VPD (V^{ant}) were defined as the weighted average of seasonal values from the year of growth (y) and the preceding four years. For season S, $q = p$ (plot) or b (block), and $X^{ant} = W^{ant}$ ($q = p$) or V^{ant} ($q = b$), the antecedent terms were defined as follows:

$$X_{y,q}^{ant} = \sum_{j=0}^{4} \sum_{s=1}^{5} w_{x,j,m} X_{y-j,S,q} \qquad \text{(Equation S6)}$$

The antecedent weights (*w*) for each variable (*X*), season (*S*), and lag year (*j*) were treated as unknown and estimated during model fitting. For each of the two groups of weights (one for W^{ant} and one for V^{ant}), we assigned a relatively non-informative Dirichlet prior that constrained the weights within each group to sum to 1 and each individual weight was constrained between 0 and 1, following Ogle et al. (2015). Antecedent weights were shared between treatments and among sites, but we allowed the overall effects of each antecedent climate term to vary by treatment, as in Equation S2.

Section S3: Supplementary Figures and Tables Describing Model Results

Table S1: A summary of covariate effects from the hierarchical linear model of annual ponderosa pine basal area increment. Columns give posterior medians, bounds of 95% credible intervals (2.5th and 97.5th percentiles), and the proportion of posterior samples with the same sign as the median, with higher proportions representing a more consistent covariate effect. Parenthetical values in the "median" column give effect sizes for covariates including an antecedent term (which have distributions that differ from scaled covariates) that can be more directly compared to other covariate effects.

**Variable**	**Treatment Status**	**2.5th %**	**Median**	**97.5th %**	**Proportion**
Available Soil Water (ASW)	Untreated	0.272	0.306 (0.212)	0.340	1
	Treated	0.104	0.170 (0.119)	0.238	1
Basal Area W/In Treatment	Untreated	-0.174	-0.226	-0.124	1
	Treated	-0.197	-0.283	-0.111	1
Sqrt(Diameter at Breast Height [DBH])	Untreated	0.453	0.503	0.553	1
	Treated	0.346	0.470	0.583	1
Vapor Pressure Deficit (VPD)	Untreated	-0.829	-0.775 (-0.363)	-0.721	1
	Treated	-1.084	-0.955 (-0.447)	-0.828	1
ASW x VPD	Untreated	0.578	0.619 (0.201)	0.663	1
	Treated	0.609	0.680 (0.221)	0.755	1
Sqrt(DBH) x ASW	Untreated	-0.015	0.009 (0.006)	0.033	0.761
	Treated	-0.127	-0.050 (-0.035)	0.020	0.920
Sqrt(DBH) x VPD	Untreated	-0.226	-0.188 (-0.088)	-0.151	1
	Treated	-0.238	-0.109 (-0.051)	0.009	0.965

Table S2: A summary of AR(1) autoregressive terms, δ terms in Equation S4, from the hierarchical linear model of annual ponderosa pine basal area increment. AR(1) terms give the influence of prior year's basal area increment (BAI; power-transformed, centered, and scaled prior to model fitting) on power-transformed BAI in the focal year. Tree-level AR(1) terms were centered around block-level terms, which were centered around site-level terms; fore brevity, only site-level terms are presented here. Columns give posterior medians, bounds of 95% credible intervals, and the proportion of posterior samples with the same sign as the median.

**Site**	**2.5th %**	**Median**	**97.5th %**	**Proportion**
Apache-Sitgreaves (AS)	0.295	0.536	0.536	1
Centennial Forest (CF)	0.249	0.427	0.427	1
Fort Valley (FV)	0.209	0.413	0.413	1
Grandview (GV)	0.183	0.523	0.523	0.999
Mt. Trumbull (MT)	0.195	0.374	0.374	1

Table S3: A summary of intercept terms, β_0 terms in Equation S3, from the hierarchical linear model of annual ponderosa pine basal area increment. Intercept terms give the predicted basal area increment (BAI; power-transformed) in each combination of site and treatment status, given values of zero (i.e., the mean in scaled variables) for other terms. Tree/treatment-level intercept terms were centered around block/treatment-level terms, which were centered around site/treatment terms; only site/treatment-level terms are presented here for brevity. Columns give posterior medians, bounds of 95% credible intervals, and the proportion of posterior samples with the same sign as the median.

**Site**	**Treatment Status**	**2.5th %**	**Median**	**97.5th %**	**Proportion**
Apache-Sitgreaves (AS)	Untreated	4.557	5.061	5.568	1
	Treated	5.209	5.743	6.285	1
Centennial Forest (CF)	Untreated	4.775	5.140	5.502	1
	Treated	5.270	5.663	6.043	1
Fort Valley (FV)	Untreated	4.449	4.859	5.280	1
	Treated	5.297	5.755	6.221	1
Grandview (GV)	Untreated	4.267	4.983	5.697	1
	Treated	4.956	5.738	6.494	1
Mt. Trumbull (MT)	Untreated	4.589	4.953	5.309	1
	Treated	5.187	5.569	5.961	1

Table S4: A summary of pairwise differences in covariate effects and intercept values between treated and untreated groups in the hierarchical regression model of annual ponderosa pine basal area increment. Positive values indicate that the treated group had a higher covariate effect or intercept estimate than the untreated group. Columns give posterior medians, bounds of the 95% credible intervals (2.5th and 97.5th percentiles), and the proportion of posterior samples with the same sign as the median, with higher proportions representing a more consistent covariate effect.

**Variable**	**2.5th %**	**Median**	**97.5th %**	**Proportion**
Available Soil Water (ASW)	-0.205	-0.136	-0.067	1
Basal Area W/In Treatment	-0.121	-0.023	0.075	0.681
Sqrt(Diameter at Breast Height [DBH])	-0.169	-0.032	0.091	0.697
Vapor Pressure Deficit (VPD)	-0.304	-0.180	-0.055	0.998
ASW x VPD	-0.009	0.061	0.132	0.957
Sqrt(DBH) x ASW	-0.141	-0.059	0.015	0.937
Sqrt(DBH) x VPD	-0.054	0.079	0.204	0.883
Treatment - AS	-0.047	0.685	1.414	0.967
Treatment - CF	-0.011	0.519	1.046	0.973
Treatment - FV	0.276	0.892	1.519	0.996
Treatment - GV	-0.279	0.751	1.802	0.927
Treatment - MT	0.090	0.621	1.139	0.988
Treatment - All Sites	0.368	0.693	1.021	1

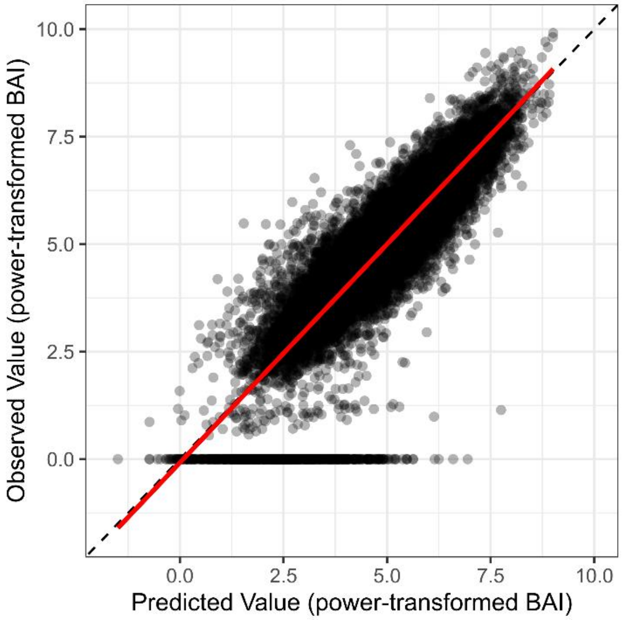

Figure S1: Model-predicted (x-axis; posterior median) vs observed (y-axis) values of power-transformed Basal Area Increment (BAI; mm^2 yr^{-1} ^0.2436712). Black dashed diagonal line is the 1:1 line, and the red diagonal line is a linear fit between observed and predicted values. The lines closely align, indicating that model covariates are effectively predicting the response without positive or negative bias.

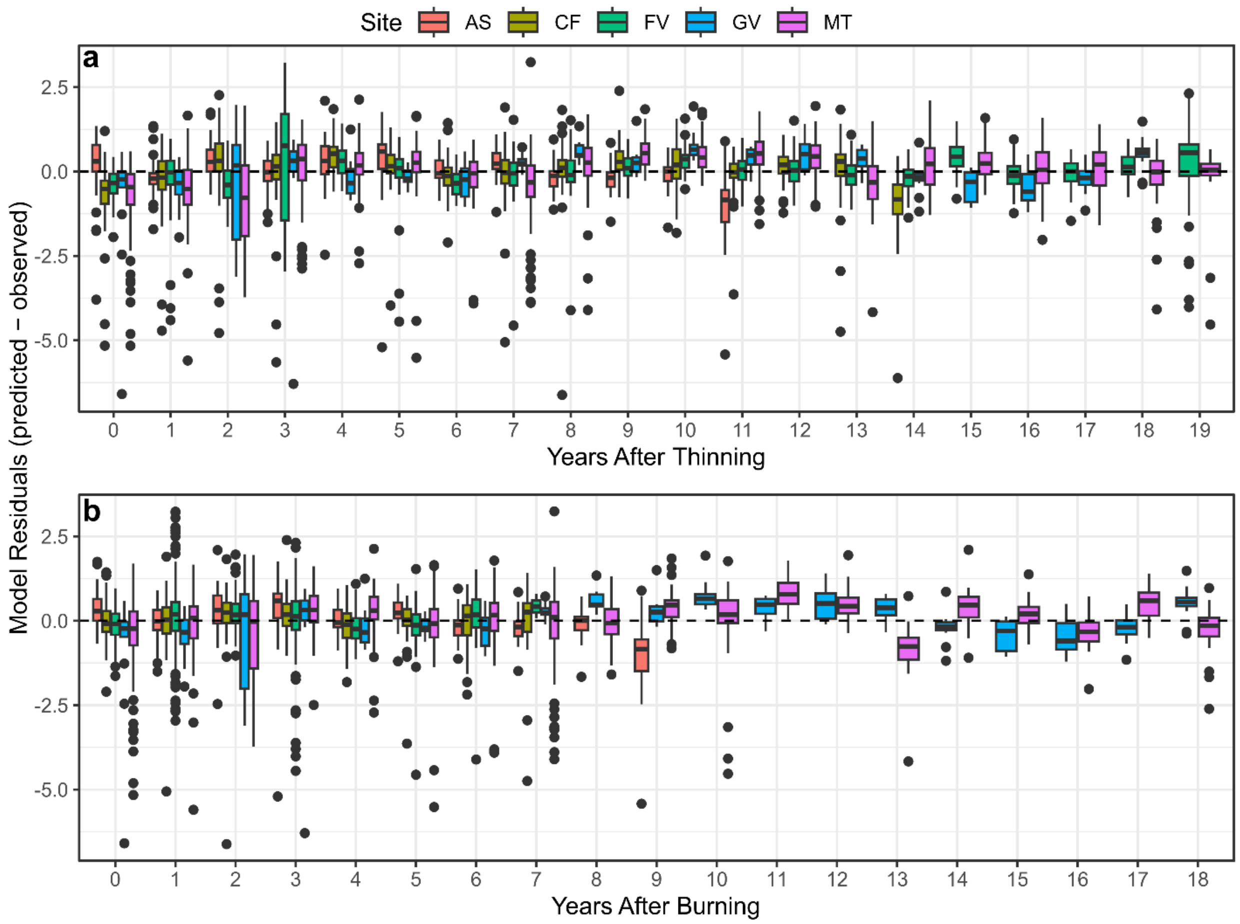

Figure S2: Model residuals (predicted minus observed values of power-transformed Basal Area Increment (BAI; $mm^2\ yr^{-1}$ ^0.2436712) as they relate to time since thinning and burning in treated trees across five experimental sites in Arizona, USA. Time since thinning and time since burning were not included in the original model structure, because only a portion of all trees on these sites were treated. Black dashes give a line at 0, where model-predicted and observed growth are identical. Values above this line indicate that growth was faster than predicted by the model, whereas values below this line indicate that growth was slower than expected. High residuals (y-axes) initially after treatment (e.g., 0-5 years after thinning), followed by neutral or negative values with greater time since thinning or burning, would indicate a positive treatment effect that wanes with time. However, there is no clear temporal pattern in model residuals either within or across sites, indicating that treatment effects were generally consistent across the study period.

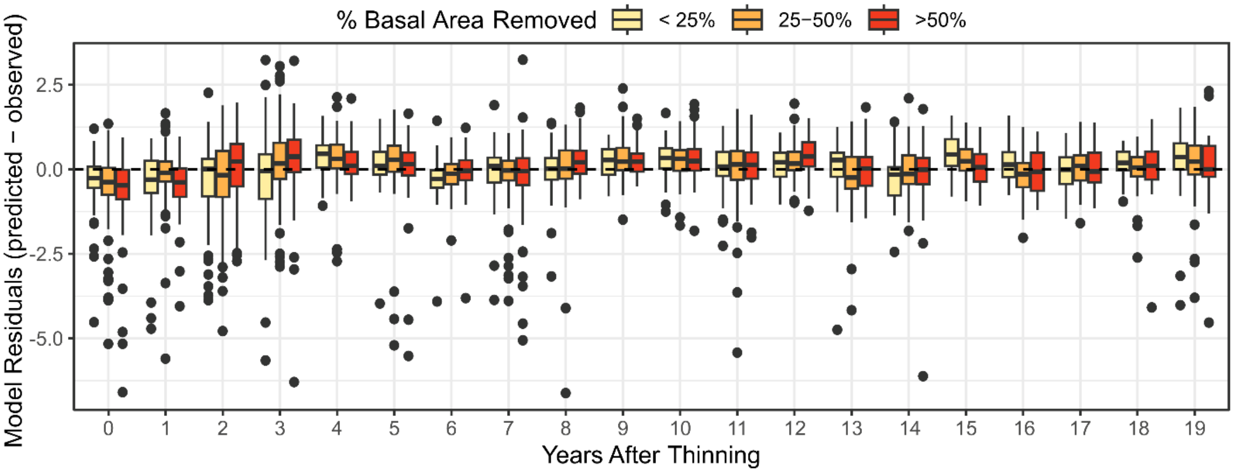

Figure S3: Model residuals (predicted minus observed values of power-transformed Basal Area Increment (BAI; $mm^2\ yr^{-1}$ ^0.2436712) as they relate to plot-level treatment intensity (i.e., percentage of initial basal area removed) across five experimental sites in Arizona, USA. Time since thinning and treatment intensity were not included in the original model structure, because only a portion of all trees on these sites were treated. Black dashes give a line at 0, where model-predicted and observed growth are identical. Values above this line indicate that growth was faster than predicted by the model, whereas values below this line indicate that growth was slower than expected. Higher residuals (y-axis values) in plots with greater basal area removal, as well as more persistent treatment effects would indicate unexplained variation due to treatment intensity. However, there is no clear pattern in model residuals across different levels of basal area removal, suggesting that these processes are already represented using covariates in the model (e.g., basal area within treatment).

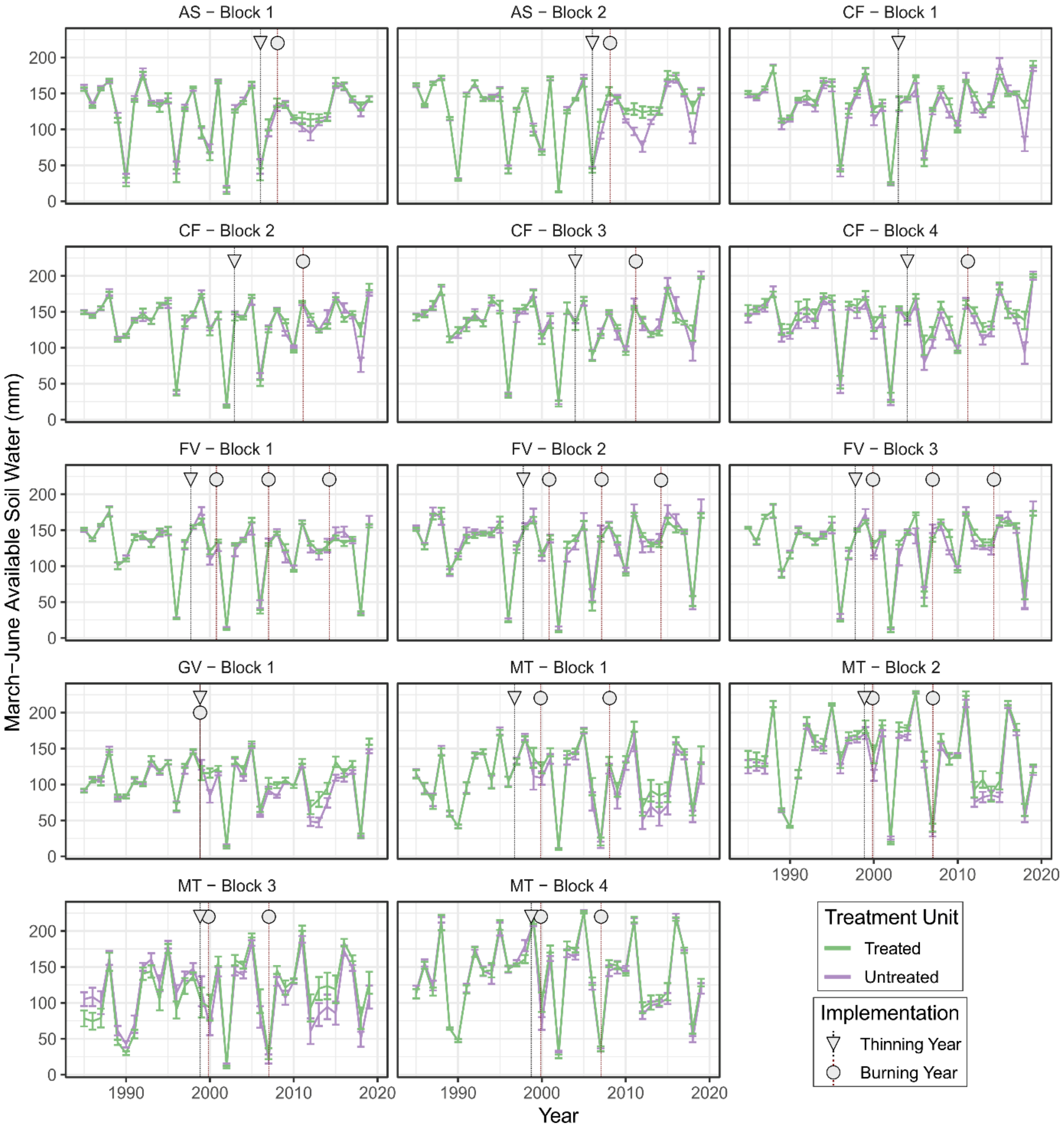

Figure S4: Modeled available soil water (ASW) (held at >-3.0 MPa) from 1985 to 2019 across experimental blocks spanning five sites in Arizona, USA. ASW values are presented for the top 1.5 m of the soil profile (a typical rooting depth for ponderosa pine) between March and June, a period that has a strong influence on tree radial growth in the our study (Figure 6 in main manuscript). Lines give mean daily values within a block and treatment unit, while error bars give 95% confidence intervals (± 1.96 * standard error of the mean). Treatment (i.e., forest thinning and prescribed burning) were implemented in different years at each block, with lines and shapes identifying the years of treatment implementation.

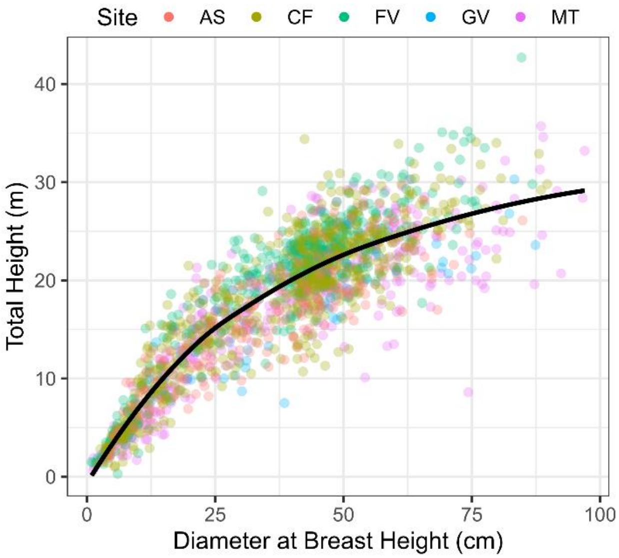

Figure S5: Relationship between diameter at breast height (DBH) and tree height based on 1,776 surveyed ponderosa pine trees (points) across our five sites (colors). The two variables were strongly correlated (Pearson's r = 0.87), indicating that DBH was a robust and general indicator of tree size. The black line gives a non-linear smooth of the data, based on a generalized additive model.

References

Allred, B. W., B. T. Bestelmeyer, C. S. Boyd, C. Brown, K. W. Davies, M. C. Duniway, L. M. Ellsworth, T. A. Erickson, S. D. Fuhlendorf, T. V. Griffiths, V. Jansen, M. O. Jones, J. Karl, A. Knight, J. D. Maestas, J. J. Maynard, S. E. McCord, D. E. Naugle, H. D. Starns, D. Twidwell, and D. R. Uden. 2021. Improving Landsat predictions of rangeland fractional cover with multitask learning and uncertainty. Methods in Ecology and Evolution 12:841–849.

Andrews, C. M., A. W. D'Amato, S. Fraver, B. Palik, M. A. Battaglia, and J. B. Bradford. 2020. Low stand density moderates growth declines during hot droughts in semi-arid forests. Journal of Applied Ecology 57:1089–1102.

Bradford, J. B., D. R. Schlaepfer, and W. K. Lauenroth. 2014. Ecohydrology of adjacent sagebrush and lodgepole pine ecosystems: The consequences of climate change and disturbance. Ecosystems 17:590–605.

Burns, R. M., and B. H. Honkala. 1990. Silvics of North America: Vol 1, conifers. Agricultural Handbook 654. US Department of Agriculure, Forest Service, Washington, D.C.

Chaney, N. W., B. Minasny, J. D. Herman, T. W. Nauman, C. W. Brungard, C. L. S. Morgan, A. B. McBratney, E. F. Wood, and Y. Yimam. 2019. POLARIS soil properties: 30-m probabilistic maps of soil properties over the contiguous United States. Water Resources Research 55:2916–2938.

Fulé, P. Z., A. J. Sánchez Meador, M. M. Moore, W. W. Covington, T. E. Kolb, D. W. Huffman, D. P. Normandin, and J. P. Roccaforte. 2022. Forest restoration treatments increased growth and did not change survival of ponderosa pines in severe drought, Arizona. Ecological Applications 32:e2717.

Gaylord, M. L., T. E. Kolb, K. F. Wallin, and M. R. Wagner. 2007. Seasonal dynamics of tree growth, physiology, and resin defenses in a northern Arizona ponderosa pine forest. Canadian Journal of Forest Research 37:1173–1183.

Huang, J. 2018. A simple accurate formula for calculating saturation vapor pressure of water and ice. Journal of Applied Meteorology and Climatology 57:1265–1272.

Ogle, K., J. J. Barber, G. A. Barron-Gafford, L. P. Bentley, J. M. Young, T. E. Huxman, M. E. Loik, and D. T. Tissue. 2015. Quantifying ecological memory in plant and ecosystem processes. Ecology Letters 18:221–235.

Peltier, D. M. P., M. Fell, and K. Ogle. 2016. Altered climatic sensitivity of tree growth after drought: Multi-species synthesis of tree-rings in the southwestern US. Ecological Monographs 86:312–326.

Rodman, K. C., T. T. Veblen, R. A. Andrus, N. J. Enright, J. B. Fontaine, A. D. Gonzalez, M. D. Redmond, and A. P. Wion. 2021. A trait-based approach to assessing resistance and resilience to wildfire in two iconic North American conifers. Journal of Ecology 109:313–326.

Sala, A., G. D. Peters, L. R. McIntyre, and M. G. Harrington. 2005. Physiological responses of ponderosa pine in western Montana to thinning, prescribed fire and burning season. Tree Physiology 25:339–348.

Schlaepfer, D. R., W. K. Lauenroth, and J. B. Bradford. 2012. Ecohydrological niche of sagebrush ecosystems. Ecohydrology 5:453–466.

Sohn, J. A., S. Saha, and J. Bauhus. 2016. Potential of forest thinning to mitigate drought stress: A meta-analysis. Forest Ecology and Management 380:261–273.

Springer, J. D., M. T. Stoddard, K. C. Rodman, D. W. Huffman, P. J. Fornwalt, R. J. Pedersen, D. C. Laughlin, C. M. McGlone, M. L. Daniels, P. Z. Fulé, M. M. Moore, B. K. Kerns, J. T. Stevens, J. E. Korb, and S. Souther. 2023. Increases in understory plant cover and richness persist following restoration treatments in Pinus ponderosa forests. Journal of Applied Ecology 61:25–35.

Steel, Z. L., M. J. Goodwin, M. D. Meyer, G. A. Fricker, H. S. J. Zald, M. D. Hurteau, and M. P. North. 2021. Do forest fuel reduction treatments confer resistance to beetle infestation and drought mortality? Ecosphere 12:e03344.

Stoddard, M. T., J. P. Roccaforte, A. J. Sánchez Meador, D. W. Huffman, P. Z. Fulé, A. E. M. Waltz, and W. W. Covington. 2021. Ecological restoration guided by historical reference conditions can increase resilience to climate change of southwestern U.S. ponderosa pine forests. Forest Ecology and Management 493:119256.

Thornton, P. E., R. Shrestha, M. Thornton, S. C. Kao, Y. Wei, and B. E. Wilson. 2021. Gridded daily weather data for North America with comprehensive uncertainty quantification. Scientific Data 8:1–17.

USGS. 2021. USGS 3D Elevation Program Digital Elevation Model.

Williams, A. P., C. D. Allen, A. K. Macalady, D. Griffin, C. A. Woodhouse, D. M. Meko, T. W. Swetnam, S. A. Rauscher, R. Seager, H. D. Grissino-Mayer, J. S. Dean, E. R. Cook, C. Gangodagamage, M. Cai, and N. G. McDowell. 2012. Temperature as a potent driver of regional forest drought stress and tree mortality. Nature Climate Change 3:292–297.
